# Improvement in an Analytical Approach for Modeling the Melting Process in Single-Screw Extruders

**DOI:** 10.3390/polym16223130

**Published:** 2024-11-09

**Authors:** Felix Knaup, Florian Brüning, Volker Schöppner

**Affiliations:** Kunststofftechnik Paderborn, Paderborn University, 33098 Paderborn, Germany; florian.bruening@ktp.uni-paderborn.de (F.B.); volker.schoeppner@ktp.uni-paderborn.de (V.S.)

**Keywords:** extrusion, melting modeling, delay zone

## Abstract

Most single-screw extruders used in the plastics processing industry are plasticizing extruders, designed to melt solid pellets or powders within the screw channel during processing. In many cases, the efficiency of the melting process acts as the primary throughput-limiting factor. If the material melts too late in the process, it may not be sufficiently mixed, resulting in substandard product quality. Accurate prediction of the melting process is therefore essential for efficient and cost-effective machine design. A practical method for engineers is the modeling of the melting process using mathematical–physical models that can be solved without complex numerical methods. These models enable rapid calculations while still providing sufficient predictive accuracy. This study revisits the modified Tadmor model by Potente, which describes the melting process and predicts the delay-zone length, extending from the hopper front edge to the point of melt pool formation. Based on extensive experimental investigations, this model is adapted by redefining the flow temperatures at the phase boundary and accounting for surface porosity at the beginning of the melting zone. Additionally, the effect of variable solid bed dynamics on model accuracy is examined. Significant model improvements were achieved by accounting for reduced heat flow into the solid bed due to the porous surface structure in the solid conveying zone, along with a new assumption for the flow temperature at the phase boundary between the solid bed and melt film.

## 1. Introduction

Single-screw plasticizing extruders are widely used in the manufacturing of polymer products, where they melt solid polymers and provide the molten material for shaping and forming processes [1]. The efficiency of this melting process is often the limiting factor for extruder throughput and significantly influences the quality of the final product. The challenge is to ensure that the polymer melts sufficiently within the screw channel to achieve proper homogenization of the melt. At the same time, overheating must be avoided to prevent material degradation. Accurate modeling is essential for optimizing extruder design and operation, which is why it has been a major research focus for decades. In addition to theoretical models that describe the melting process based on fundamental assumptions, numerous models utilize numerical flow simulations to explain melting behavior. In these simulations, no specific melting mechanism is initially assumed. Instead, the system is described using the conservation equations for mass, momentum, and energy. The core idea of most studies is the calculation of the melting process through a representative material property, such as heat capacity or viscosity. Early simulations described in the literature were based on the finite element method [2,3,4]. However, in recent years, the finite volume method (FVM) has gained significance, particularly due to the continuous increase in computational power and availability. Examples of computational fluid dynamics (CFD) simulations based on the FVM can be found in [5,6,7,8,9,10,11]. A relatively new approach to simulating the melting process is to couple CFD simulations with the discrete element method (DEM) to describe the flow of the pellet particles, as described in [12]. Despite the improvements in numerical simulation models, the use of these simulations still requires specific expertise and is associated with significant computational resources.

The main advantage of theoretical models with a predefined melting mechanism lies in their simpler handling and the typically much faster generation of results, especially when dealing with analytically solvable systems. To provide a better understanding, the following sections present an overview of the historical development of mathematical–physical melting models for single-screw extruders.

**Figure 1:**
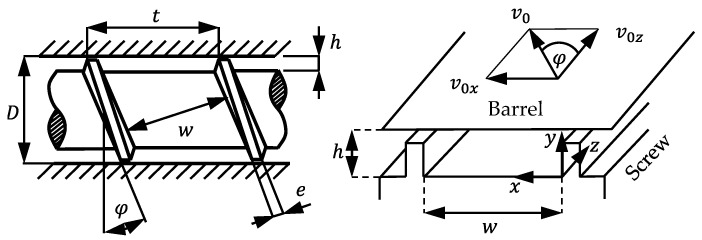
Unwrapped view and coordinate system for the mathematical treatment of flow in the screw channel.

A fundamental concept for almost all theoretical modeling approaches to the melting process is a straight channel, which is formed by an imaginary unwrapping of the screw. The unwrapped channel and the fixed coordinate system are shown in Figure 1.

Here, D is the barrel diameter, t is the pitch, φ is the helix angle, h is the channel depth, w is the channel width, e is the flight width, and $v_{0}$ is the barrel circumferential speed.

The first systematic investigations of the melting process in single-screw extruders were conducted by Maddock in 1959 [13] and later confirmed by Street [14]. In these experiments, the extruder was abruptly stopped from a steady-state condition, then cooled, and the screw was removed. The solidified polymer was then unwound from the screw, and cross-sectional samples were taken. By adding 3–5% colored pellets, Maddock was able to distinguish between the molten and solid portions of the cross-sectional samples. He observed that a thin melt film formed along the barrel surface and a melt pool developed on the active screw flight. As the polymer progressed along the screw channel, the melt pool increased mainly in width until the solid material was completely melted [13,14].

**Figure 2:**
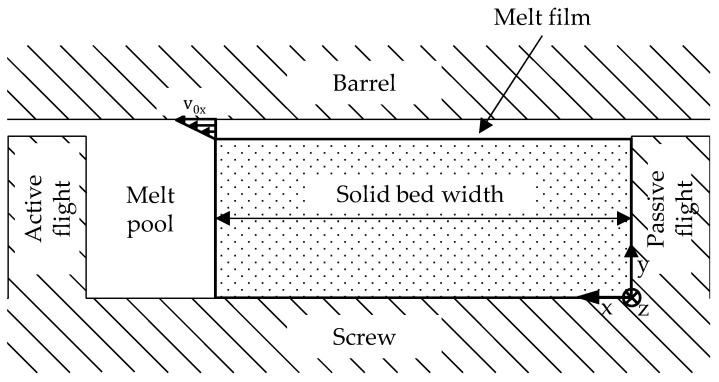
Melting model according to Tadmor [15].

Maddock’s experimental observations were first theoretically described by Tadmor et al. in 1966 [15]. According to Talmor’s model, the melting process occurs exclusively between the solid bed, which flows through the melting channel at a constant speed, and the barrel, where a melt film with a constant thickness is formed. At the phase boundary between the melt film and the solids, the material’s melting temperature is present. The film is transported by drag flow due to the barrel’s circumferential velocity in the transverse channel direction, $v_{0x}$, across the melting channel into the melt pool. The dominant mechanisms in the melt film are heat conduction in the y-direction and viscous dissipation due to shearing. Heat convection is neglected in this model. Furthermore, Tadmor assumes constant physical properties for both the solid and the melt and neglects the leakage flow over the flights. The model described by Tadmor is depicted in Figure 2 [15].

The target value in Talmor’s model is the solid bed width as a function of the channel length coordinate z. This can be calculated from a mass balance, assuming a constant melt film thickness along the channel’s longitudinal direction. A comparison with Maddock’s experimental investigations showed that the melting rate is overestimated at the beginning of the melting zone and underestimated at the end of the melting zone [15]. Tadmor suggested that the accelerated melting rate toward the end of the melting zone is likely due to the breaking of the solid bed [15].

Following extensive experimental investigations of their own, Tadmor et al. made modifications to improve the accuracy of the model [16]. These modifications included accounting for the shear-thinning behavior of polymers, assuming a non-linear velocity and temperature profile within the melt film, and considering convection at the phase boundary between the solid bed and the melt film. Unlike the original model, the modified version could only be solved numerically, but it led to significantly better agreement with experimental results. In a further adaptation of the model, Tadmor also accounted for the effects of screw channel curvature and screw clearance [17]. Hinrichs and Lilleleht also described the effects of these two influencing factors through their own modification of the Tadmor model [18]. A detailed discussion of the effects of various process and geometric parameters, as calculated from the modified Tadmor model, can be found in [17].

Furthermore, Tadmor observed a so-called delay in melting in his own experiments. This delay was partly attributed to insufficient barrel temperature and partly to the initial melt, generated by heat conduction, penetrating into voids within the solid bed. He defined the region between the end of the solid conveying zone and the beginning of melt pool formation as the “delay zone”. This zone is crucial for calculating the melting length, as it can account for up to 20% of the total length [19]. In [19], he presented a model that calculates the melt film thickness in the delay zone based on heat conduction and viscous dissipation. The effect of melt penetrating into the solid bed was later confirmed through borescope investigations [20].

A critical discussion of Talmor’s original model was conducted by Chung [21]. He proposed a total of six improvements to the model, some of which had already been incorporated into Talmor’s modified versions. His first point of criticism was the assumption of a constant temperature profile in the solid bed, which led to an inaccurate prediction of the melting process. The heating of the solid bed, especially toward the end of the melting zone, results in an increased melting rate, which must therefore be considered in the modeling. He suggested treating the height of the solid bed as finite and equal to the depth of the screw channel, which would better represent the increased melting rate toward the end of the melting zone. Additionally, he criticized the assumption of a constant solid bed density, as the solid bed in reality becomes compacted due to the increasing pressure in the screw channel. Chung therefore proposed a linear approximation of the density from the bulk density at the beginning of the melting zone to the compacted melt at the end of the melting zone. He also criticized the assumption of a constant solid bed velocity, which was arbitrarily assumed and should be verified through experimental and theoretical studies. The fourth point of criticism was the assumption of a constant melt film thickness. In a steady state, the continuous scraping of the melt from the barrel would result in a variable melt film thickness, which should be taken into account in the modeling. The remaining criticisms included the failure to consider the screw clearance, as well as the neglect of the channel curvature and the resulting larger contact area between the solid bed and the melt [21].

In 1971, Donovan achieved a similar analysis as Chung regarding the heating of the solid bed and accounted for a temperature increase within the solid bed [22]. Furthermore, he argued that the assumption of a constant solid bed velocity was inaccurate. He extended Talmor’s model by introducing a material-dependent Solid Bed Acceleration Parameter (SBAP), which leads to a uniform acceleration of the solid bed along tapering channel cross-sections. Additionally, he described a melt film thickness that increases linearly from the solid bed toward the melt pool. The increasing melt film thickness in the direction of the drag flow had already been described earlier by Vermeulen et al. [23]. However, for the sake of mathematical manageability, Donovan assumed a constant melt film thickness where necessary [22].

In the following years, Edmondson and Fenner [24] built upon Donovan’s findings and also assumed a variable solid bed velocity for their model. The calculation of the solid bed velocity was based on a force balance. Additionally, they extended the melting model by incorporating a mathematical description of a melt film that forms between the solid bed and the screw surface. Later, Cox and Fenner expanded this model [25] by integrating findings from experimental investigations aimed at determining the solid bed velocity.

**Figure 3:**
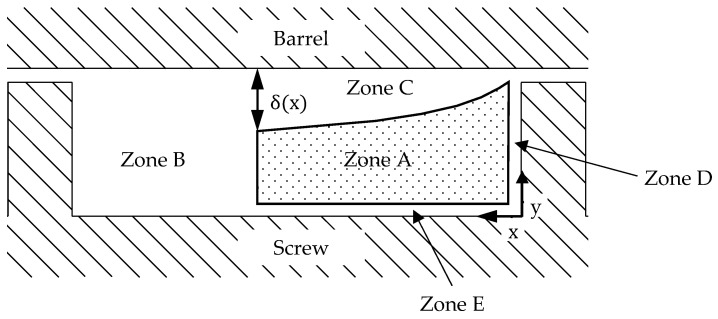
Five-zone melting model according to Pearson [27].

In [26], Pearson theoretically demonstrated that the assumption of a constant melt film thickness would lead to an unrealistically high-pressure gradient along the channel, thereby confirming the variable melt film thickness previously described by Donovan and Vermeulen. Building on his findings, Pearson et al. described a five-zone model for calculating the melting process in single-screw extruders [27]. The model is depicted in Figure 3. In addition to the increasing melt film thickness δ(x) toward the melt pool (Zone C), it also accounts for the melt films on the passive flight (Zone D) and at the screw root (Zone E). The assumptions made in this model are more realistic than in most other models, as the melt films on the screw have also been observed in experiments. However, this also makes the model significantly more complex and computationally demanding.

In 1982, Mount, Watson, and Chung presented an analytically solvable model for the melting process [28,29]. They utilized dimensionless differential equations to find approximate solutions through limit analyses, addressing equations that were otherwise unsolvable. By assuming temperature-dependent viscosity and a negligible Brinkman number, they demonstrated the proportionality $\delta \sim \sqrt{x}$ for the melt film thickness δ as a function of location x. The analytically calculated melting rates were tested against experimental investigations for several semi-crystalline and three amorphous thermoplastics using a screw simulator, yielding good results [28].

Also in 1982, Fukase published a melting model following numerous investigations of the melting process [30]. Contrary to Tadmor’s modeling, it was demonstrated that the solid bed not only decreases in width but also significantly in thickness. Fukase explicitly studied the influence of solid bed velocity by measuring the distance between colored pellet lines in the solid bed of the unwound channel. These lines were created by continuously feeding colored masterbatch into the hopper through a thin tube. Two boundary cases emerged for the behavior of the solid bed velocity: if the solid bed melts sufficiently quickly in both width and thickness as the channel depth decreases, the solid bed velocity remains constant. However, if the channel depth decreases too early, causing a “dam-up” of the solid bed, the melt film on the barrel disappears, and the solid bed velocity increases proportionally as the channel depth decreases.

In 1989, Rauwendaal introduced another model for calculating the melting behavior in single-screw extruders [31,32]. The described model remains analytically solvable while accounting for shear-thinning behavior and the temperature dependence of viscosity. Although the model is not compared with experimental data, the analytical description allows for a precise discussion of the effects of various influencing factors. Based on his model, he examined the different influences of process and geometry parameters on the melting process, arriving at conclusions similar to those of Tadmor in [17].

**Figure 4:**
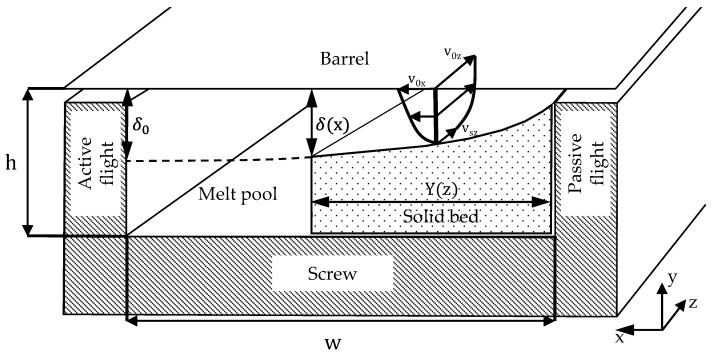
Modified Tadmor model according to Potente [33].

Also in 1989, Potente presented an approximate analytical solution to the Tadmor model, considering the temperature-dependent shear-thinning behavior of polymer melts with a variable melt film thickness [33]. The described model is depicted in Figure 4.

Potente demonstrated that when plotted on a double logarithmic scale, there is a good approximation of a linear relationship between the melt film thickness at the barrel δ(x) and the solid bed width Y. Accordingly, the melt film thickness at the barrel δ(x) as a function of the position coordinate x, the channel width w, the melt film thickness $\delta_{0}$ at the position x=w, and the contour exponent c follows the relationship given in the following Equation [33]:

(1) $$\frac{\delta (x)}{\delta_{0}}={\left(\frac{x}{w}\right)}^{c}$$

Furthermore, Potente theoretically considered the possible acceleration and compression of the solid bed in tapered sections. Here, the solid bed width as a function of the channel length coordinate z is calculated using the following equation [33]:

(2) $$Y=\left\{\left[\frac{\Psi_{1}\left(1-c^{\prime }\right)\left(1-\Psi_{S}\right)\pi }{\beta \left[\left(1-c^{\prime }\right)\left(1-a\right)+a\right]}\right]u^{a}+{\left[\left(Y_{1}^{1-c^{\prime }}-\frac{\Psi_{1}\left(1-c^{\prime }\right)\left(1-\Psi_{S}\right)\pi }{\beta \left[\left(1-c^{\prime }\right)\left(1-a\right)+a\right]}\right)\cdot \frac{1}{u^{\left(1-c^{\prime }\right)\left(1-a\right)}}\right]}^{\frac{1}{1-c^{\prime }}}\right\}$$

In this equation, Y is the dimensionless solid bed width, $\Psi_{1}$ is the dimensionless melt film thickness, $c^{\prime }$ is the contour exponent considering the leakage flow over the flight of the melt film at the barrel, π is the melting rate, *u* is the dimensionless height of the solid bed, and β is a slope parameter used to describe *u* as a function of the dimensionless channel length coordinate ζ. The parameters *a* and u are defined as follows:

(3) $$\frac{\rho_{S}v_{sz}}{\rho_{S0}v_{sz0}}=u^{-a}={\left(\frac{h_{s}}{h_{s0}}\right)}^{-a}={\left(1-\beta \zeta \right)}^{-a}$$

In this equation, $\rho_{S}$ is the density of the solid bed, $v_{sz}$ is the velocity of the solid bed in the channel longitudinal direction z, and $h_{s}$ is the height of the solid bed. The values indexed with “0” represent the state at the beginning of the considered section. For a>0, the acceleration of the solid bed outweighs the density change; for a<0, compression of the solid bed occurs. Due to the lack of experimental data, the assumption a=0 has been used so far, meaning that the density changes and the acceleration of the solid bed balance each other out. Using Potente’s analytical model, shorter melting lengths are calculated compared to the models of Tadmor and Chung, which corresponds to better agreement with experimental investigations [33].

In contrast to most other melting models, Potente’s model also accounts for the delay zone, as previously discussed by Tadmor, enabling the calculation of the entire melting length. According to Potente, the melt film thickness in the delay zone increases due to heat conduction from the barrel and viscous dissipation until the melt film reaches a necessary thickness. The necessary melt film thickness is defined as the average melt film thickness during the melting process at the current location in the screw channel. Once this thickness is reached, the delay zone ends and melt pool formation begins. Unlike Tadmor, Potente assumed a reduced heat flux into the solid bed when calculating the melt film thickness in the delay zone, as the solid bed in this area is porous on the surface. However, a precise definition of surface porosity and verification against experimental data was not provided [33].

Previous investigations have demonstrated the development of several theoretical models describing the melting process in single-screw extruders. Analytical solvable models, in particular, offer the primary advantage of ease of use. Among these, the modified Tadmor model by Potente provides a comprehensive description of the melting process, including the consideration of the delay zone. However, this model has not yet been validated through extensive experimental investigations. Additionally, certain parameters within the model remain insufficiently defined due to a lack of experimental data. These include, in particular, suitable assumptions for the surface porosity within the solid conveying zone and for the parameter a, which describes the dynamics of the solid bed.

The investigations presented in this paper aim to validate and adjust the Potente model based on experimental data, ensuring that the results from experimental studies are accurately represented while preserving the advantages of an analytically solvable model. This study specifically focuses on determining the appropriate temperature at phase boundaries, describing surface porosity within the delay zone, and validating suitable assumptions for solid bed dynamics in compression zones, particularly concerning the parameter a.

## 2. Materials and Methods

The following section details the materials, machines, and screws used for the experimental investigations. Additionally, it outlines the methodologies employed to identify the location of melt pool formation and to characterize the melting profiles based on the experimental data.

### 2.1. Materials

The materials examined in this study consist of a semi-crystalline high-density polyethylene (HDPE) of the type Hostalen CRP 100 RESIST CR Natural, supplied by LyondellBasell Industries (Rotterdam, The Netherlands), and an amorphous polystyrene (PS) of the type PS 124N, which is transparent in color and supplied by INEOS Styrolution (Frankfurt am Main, Germany). To differentiate between the solid bed and melt pool in the sectioned samples, a 5 wt.% addition of black-colored HDPE granules was incorporated, while a similar percentage of white-colored PS pellets was used for the polystyrene samples. Additionally, to investigate the impact of varying surface porosities on the length of the delay zone, pellets with average diameters of 1 mm and 2 mm were examined alongside the standard 3 mm pellets. These pellets were manufactured by MAAG Germany GmbH (Grossostheim, Germany) using the Pearlo underwater pelletizing system.

### 2.2. Extruders and Screws

Two extruders were employed for the investigations: an ESE 1-30-33 extruder with a 30 mm barrel diameter from ESDE Maschinentechnik GmbH (Bad Oeyenhausen, Germany) and an RH-034-45-28D/HS extruder featuring a 45 mm barrel diameter from Reifenhäuser GmbH & Co. KG Maschinenfabrik (Troisdorf, Germany). Both machines are housed in the laboratories of Kunststofftechnik Paderborn (KTP) at Paderborn University.

To investigate the delay zone length as a function of different channel volumes, experiments were conducted using a 45 mm diameter screw and a 30 mm diameter screw. Additionally, the solid bed dynamics were studied using two available 30 mm diameter screws with different compression zone lengths. In total, three different three-zone screws were used for these investigations. The geometries used are specified in Table 1.

### 2.3. Investigation Method

In addition to the method described by Maddock in the introduction, other methods for investigating the melting behavior in single-screw extruders are discussed in the literature. A selection of these methods can be found in the following references [20,34,35,36]. However, the screw-pull experiments described by Maddock represent the only method that enables a comprehensive investigation of the melting process independent of the position of measuring instruments. For this reason, this method is also used in these investigations. Preliminary studies indicated that the direct addition of 0.1 wt.% carbon black effectively distinguished between the solid bed and the melt pool but significantly reduced throughput compared to pure pellets. Therefore, material mixtures containing 5 wt.% colored pellets were utilized in these investigations, which did not result in any changes in throughput.

#### 2.3.1. Determination of the Delay Zone Length

**Figure 5:**
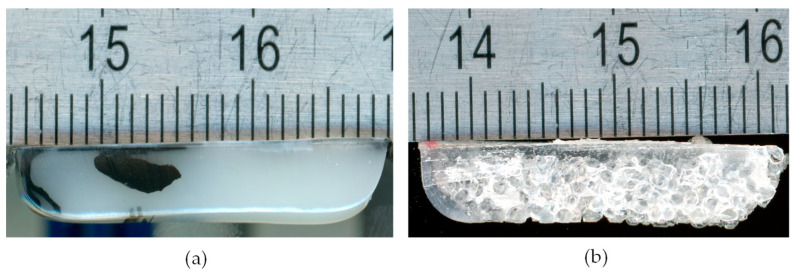
Cross-sectional samples at the location of melt pool formation. (**a**) HDPE cross-section, melt pool formation marked by material mixing of natural-colored and black material on the left edge. (**b**) PS cross-section, melt pool formation marked by material mixing of transparent and natural-colored material on the left edge. Scale in millimeters.

To determine the length of the delay zone up to the point of melt pool formation, the solidified melt is removed from the channel. Cross-sectional samples are then taken, starting from the hopper front edge, until a melt film is first observed on the active channel flight. Examples of such cross-sectional samples are shown in Figure 5.

The position where the melt pool was first observed is then measured. Since the chosen investigation method does not allow for an exact determination of the point of melt pool formation, intervals of 0.5 L/D were established along the screw channel. The interval in which the melt pool was first detected is then indicated.

#### 2.3.2. Determination of the Melting Profile

**Figure 6:**
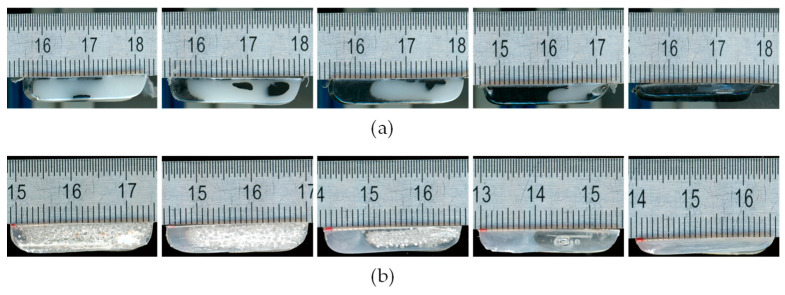
Cross-section samples along the melting zone. (**a**) HDPE. (**b**) PS. Scale in millimeters.

To determine the melting profile, additional cross-sectional samples were taken from the channel and analyzed. The main goal was to determine the melting profile at characteristic points along the screw channel. After identifying the point of melt pool formation, the end of the melting process was first determined. Subsequently, samples were taken at the beginning of the compression zone, in the middle of the compression zone, and at the end of the compression zone. Depending on the test conditions, additional samples were taken between these points and in the metering zone. The samples, shown in Figure 6, were then scanned and digitally measured using the image processing software ImageJ 1.54g.

The average width of the solid bed and the proportion of the solid bed in the total cross-section were measured. This allowed the melting profile to be determined both in terms of the solid bed width and the solid bed area fraction.

### 2.4. Experimental Design

The experimental investigations aim to create a data set that can be used to describe the effects of various process parameters on the melting behavior. Utilizing this data set, the study will thoroughly examine both the length of the delay zone and the solid bed dynamics within the compression zones. To investigate these specific aspects of the melting zone, two experimental plans were developed. The upper-speed limit in both plans was selected to ensure that the PS124N in the ESE 1-30-33 extruder just barely fails to fully melt within the processing length, while the lower speed limit was appropriately adjusted to encompass a speed range typical for extrusion processes. Additionally, the barrel temperature was varied by ±30 °C around the manufacturer’s recommended setting of 200 °C. To assess the impact of pellet size, three different pellet diameters were available and utilized for each material.

#### 2.4.1. Investigation of the Delay Zone Length

For the investigation of the delay zone length, five influencing factors were selected from the wide range of variables for detailed examination. Table 2 presents the experimental plan, which was developed using a central composite design. In this design, pellet diameter was varied across three factor levels, while the factor levels marked α with represent the star points, with α set to 1.5 for this experimental setup.

The experimental plan was carried out for the two previously introduced materials and both extruders. To verify reproducibility, four repetitions of the central point were conducted for both materials on the ESE 1-30-33 extruder with Screw 2. Screws 1 and 2 were utilized in this experimental plan to assess the impact of different channel volumes and, consequently varying bulk densities on the delay zone length. Screw 3, being geometrically identical to Screw 2 in the feed zone, was excluded from these investigations. In total, 54 experimental results were generated to examine the delay zone length, with melting profiles determined for each case.

#### 2.4.2. Investigation of Solid Bed Dynamics

To analyze the influence of different compression gradients on the solid bed dynamics and, consequently, on the melting profile, an additional experimental plan was developed. A central composite design, as illustrated in Table 3, was implemented using the ESE 1-30-33 extruder with Screws 2 and 3 for the two previously mentioned materials.

Back pressure was adjusted using a throttle die, where the outlet cross-section was modified by adjusting a bolt. A low back pressure corresponded to a bolt insertion depth of 0 mm, a medium back pressure of 7.5 mm, and a high back pressure of 15 mm. The barrel temperature was set to 200 °C for all investigation points. To verify reproducibility, some of the experimental points were randomly repeated, resulting in a total of 71 experimental data points being evaluated.

### 2.5. Modeling

The fundamental model used for the investigations in this paper is the model described by Potente in [33]. Based on the previously described experimental investigations, parameters that have been insufficiently defined thus far will be empirically adjusted to achieve the best possible agreement between the model predictions and the experimental data. The software REX 17.1 (computer-aided extruder design, developed at KTP) was used for the calculations, which incorporates Potente’s model. For the calculation of the melting profile, the experimentally determined throughput was specified in each case. The subsequent section details the adjustments made to the model based on the experimental data, beginning with the selection of flow temperature. Preliminary studies indicated that utilizing the glass transition temperature ${(T}_{g})$ for amorphous thermoplastics and the Differential Scanning Calorimetry (DSC) peak temperature $\left(T_{m}\right)$ for semi-crystalline thermoplastics results in a significant underestimation of the melting length.

#### 2.5.1. Definition of the Flow Temperature

**Figure 7:**
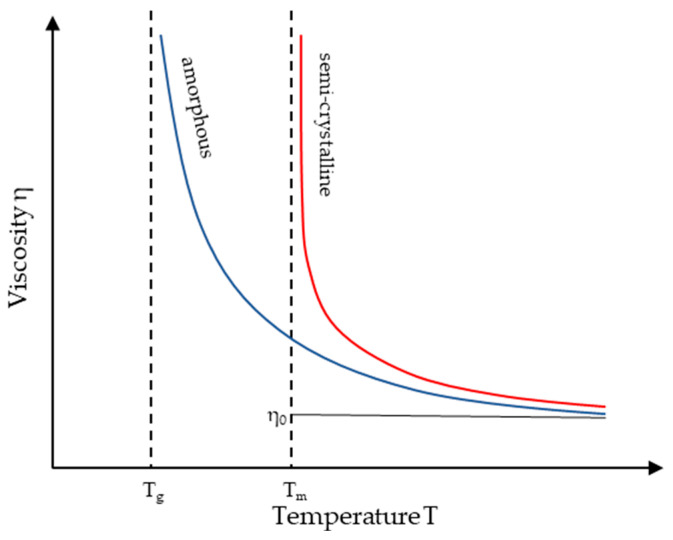
Temperature dependence of viscosity [37,38].

A key assumption of the model is the interface between the solid bed and the melt film, characterized by a sharp transition at the melting temperature $T_{m}$ for semi-crystalline thermoplastics, or $T_{g}$ for amorphous thermoplastics, as these are defined as the melting temperatures of the materials [33]. However, these temperatures do not represent a transition into a flowing state, as both semi-crystalline and amorphous thermoplastics are not sufficiently low in viscosity to flow at these temperatures. This is illustrated in Figure 7.

Amorphous thermoplastics only achieve sufficient flowability well above their glass transition temperature. In [39], this flow temperature for two amorphous thermoplastics was determined through model fitting to experimental data at approximately 55 °C above $T_{g}$. In [40], five regions of viscoelastic behavior are described for atactic polystyrene, determined by the relaxation modulus $E_{r}$. The rubbery flow state begins at approximately 57 °C above the glass transition temperature, while the purely viscous state only begins at a temperature of around 77 °C above the glass transition temperature. Given the challenge in precisely determining the exact flow temperature of amorphous thermoplastics, which is undoubtedly considerably higher than $T_{g}$, this study adopts a simplified assumption. The flow temperature for amorphous thermoplastics is set to 50 °C above the glass transition temperature, which is cited as a typical value in [41]. The glass transition temperature of the PS 124N used in these investigations was determined via DSC in the laboratories of KTP at Paderborn University to be 92.3 °C. Consequently, the flow temperature for modeling is set at 142.3 °C. All DSC measurements were conducted using the DSC3 STARe system from Mettler Toledo (Columbus, OH, USA) in accordance with DIN EN ISO 11357-3 [42].

**Figure 8:**
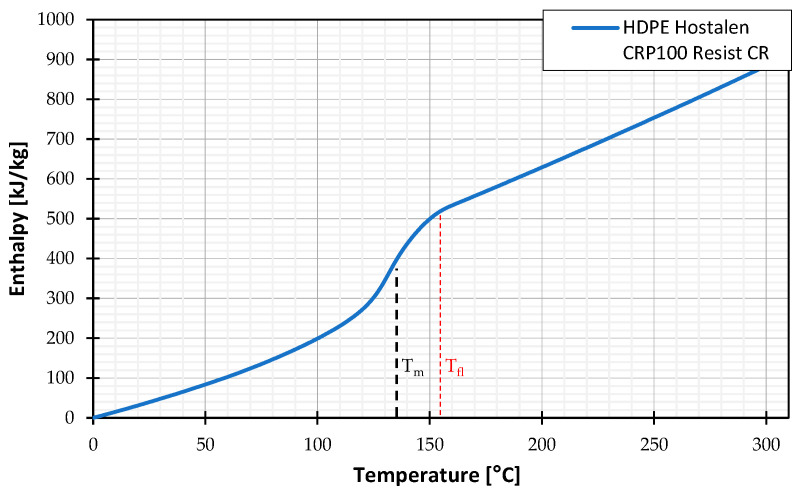
Determination of the assumed flow temperature of HDPE Hostalen CRP100 Resist CR based on the enthalpy curve, determined from a DSC measurement.

The crystalline melting temperature is also typically determined using DSC. The melting point is defined at the endothermic peak of the heat flow curve during heating. However, this peak does not represent the point at which all crystallites have melted, but the point where the melting rate of the crystallites is at its highest. As shown in Figure 7, the material remains too viscous at $T_{m}$ to flow. By recognizing that semi-crystalline thermoplastics do not possess a distinct melting point but instead exhibit a melting range, this study does not assume the flow temperature to be at $T_{m}$, which was determined by DSC measurement to be 133 °C. Instead, the flow temperature is set at the end of the melting range, as determined from the enthalpy curve and established at 152 °C for this study. The determination of the flow temperature based on the enthalpy curve of HDPE Hostalen CRP100 Resist CR is presented in Figure 8.

#### 2.5.2. Modeling of the Delay Zone Length

The basic concept in describing the melting length according to Potente is the assumption of a gradually increasing melt film thickness δ. The point of melt pool formation is reached once this melt film thickness reaches a critical value. The necessary melt film thickness is considered to be the average melt film thickness during the melting process at the current position in the screw channel [33,43]. To calculate the melt film thickness, it is necessary to balance the heat fluxes at the phase boundary interface. The heat flux ${\dot{q}}_{sb}$ into the solid bed can be calculated as follows [33]:

(4) $$\begin{array}{c} {\dot{q}}_{sb}=\frac{\lambda_{S}\left(T_{fl}-T_{s}\right)}{\sqrt{\pi a_{s}}}\sqrt{\frac{v_{sz}}{\varepsilon_{S}Z}}\exp\left\{-{\left[\frac{k_{1}}{4}\frac{\rho_{m}}{\rho_{s}}\left(\frac{v_{0z}}{v_{sz}}-1\right)\sqrt{\frac{v_{sz}}{a_{s}\varepsilon_{F}Z}{\bar{\delta }}_{AS}}\right]}^{2}\right\}+\\ \frac{k_{1}}{4}\rho_{M}\left(\frac{v_{0z}}{v_{sz}}-1\right)\Delta h_{s}\frac{{\bar{\delta }}_{AS}v_{sz}}{\varepsilon Z}\left\{1+\mathrm{er}f\left[\frac{k_{1}}{4}\frac{\rho_{m}}{\rho_{s}}\left(\frac{v_{0z}}{v_{sz}}-1\right)\sqrt{\frac{v_{sz}}{a_{s}\varepsilon_{F}Z}{\bar{\delta }}_{AS}}\right]\right\} \end{array}$$

A detailed derivation can be found in [33]. In this equation, $\lambda_{S}$ describes the thermal conductivity of the solid, $T_{fl}$ is the flow temperature at the phase boundary, $T_{s}$ is the temperature at the center of the solid bed, π describes the melting rate, $a_{s}$ is the thermal diffusivity of the solid, $v_{0z}$ is the circumferential velocity of the barrel in the z-direction, $v_{sz}$ is the solid bed velocity in the z-direction, Z is the length of the interval, $\rho_{m}$ is the density of the melt, $\rho_{s}$ is the density of the solid bed, $k_{1}$ is a parameter describing the temperature-dependent viscosity, $\Delta h_{s}$ represents the enthalpy difference in the solid up to the melting temperature, and ${\bar{\delta }}_{AS}$ is the average melt film thickness at the point of melt pool formation. ε describes the portion of the surface that is not in contact with the barrel, while $\varepsilon_{S}$ describes the portion that is in contact with the barrel surface. Consequently, the following equation applies [33]:

(5) $$\varepsilon =1-\varepsilon_{S}$$

**Figure 9:**
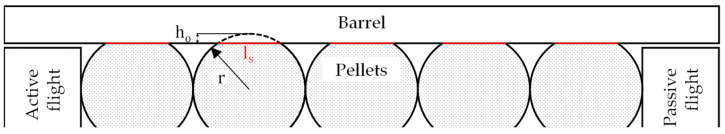
Schematic illustration of the reduced contact area at the beginning of the screw channel.

A precise method for calculating the surface porosity is not provided in [33]. Therefore, the following develops an approach based on the bulk porosity present in the channel. The initial state of the screw channel, where no material has yet melted, is schematically illustrated Figure 9.

**Figure 10:**
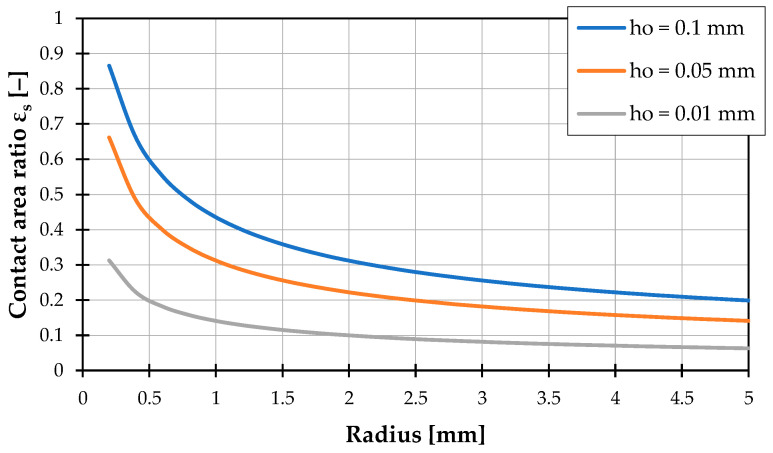
Dependence of the contact area ratio on the circle radius for different assumed overlap heights.

As depicted in Figure 9, the pellets have a limited contact area with the barrel, especially at the beginning of the screw channel. In this zone, heat input from the barrel primarily occurs through the contact area highlighted in red. In the schematic illustration, pellets are represented as circles. Based on this model concept, the contact area with the barrel can be determined by calculating the length of the chord, given a radius r and an assumed overlap height $h_{o}$. This calculation subsequently determines the contact area between the pellets and the barrel. Figure 10 demonstrates that the calculated contact area is highly dependent on pellet size and the assumed overlap height. Since the overlap is a conceptual model that does not exist in reality, $h_{o}$ is illustrated here with arbitrary examples of 0.01 mm, 0.05 mm, and 0.1 mm. The exact contact area is extremely difficult to determine theoretically. However, it can be observed that the contact area increases as the pellet diameter decreases.

Additionally, within the delay zone, both the changing contact area due to increasing pressure and pellet melting, as well as the effect described in [19]—where the initially formed melt penetrates the solid bed without immediately forming a film—occur simultaneously. The combined influence of these effects further complicates the calculation. Nevertheless, to account for the effect of the porous surface on heat transfer, a simplified approach based on the channel’s bulk porosity has been adopted. Unlike surface porosity, the bulk porosity $\Phi_{channel}$ can be easily calculated from the bulk density $\rho_{b,channel}$ present in the channel and the solid’s density $\rho_{0}$ [44].

(6) $$\Phi_{channel}=1-\frac{\rho_{b,channel}}{\rho_{0}}$$

The bulk density in the channel can be determined according to [45], based on the standard bulk density $\rho_{b,0}$ using the ISO 60 method [46], the channel depth h, the channel width w, and the pellet diameter $d_{p}$. The equation for calculating the bulk density in the channel is as follows [45]:

(7) $$\rho_{b,channel}=\rho_{b,0}\cdot \frac{\left[\left(\frac{h}{d_{p}}\right)-1\right]\cdot \left[\left(\frac{w}{d_{p}}\right)-1\right]+\frac{1}{\sqrt{2}}\cdot \left[\left(\frac{h}{d_{p}}\right)\right.+\left.\left(\frac{w}{d_{p}}\right)-2\right]+\frac{1}{2}}{\left[\left(\frac{h}{d_{p}}\right)-1\right]\cdot \left[\left(\frac{b}{d_{p}}\right)-1\right]+\left.\left(\frac{h}{d_{p}}\right)\right.+\left.\left(\frac{w}{d_{p}}\right)\right.-1}$$

The calculation of surface porosity, or the contact area between the pellets and the barrel, is now derived from the bulk porosity using the following approach:

(8) $$\varepsilon_{s}={\left(1-\Phi_{Channel}\right)}^{E}$$

Here, E is an empirically determined correction exponent that describes the difference between bulk porosity and surface porosity in the delay zone using a simplified equation. This exponent specifically accounts for the dependency on pellet diameter, as illustrated in Figure 10. The exponent was adjusted to fit the experimental data using the method of least squares, with the best fit found for E=1.67.

#### 2.5.3. Modeling of Solid Bed Dynamics

The solid bed dynamics within compression zones are theoretically modeled using the previously described exponent a, which was defined in Equations (2) and (3). Given the challenges associated with measuring both the solid bed velocity and the density of the solid bed during the extrusion process, an empirical approach is adopted to account for the effect of solid bed dynamics on the melting process. The primary objective is to accurately predict the end of melting, which is crucial for effective process design. The investigation is conducted over a range from a=−1, which represents compression or a deceleration of the solid bed proportional to the channel depth, to a=1, meaning an acceleration of the solid bed proportional to the change in channel depth. The comparison between the model and experimental investigations is carried out for the parameters a=−1, a=−0.5, a=0, a=0.5 and a=1.

## 3. Results

This chapter presents the findings from the experimental investigations and the corresponding model comparisons. The analysis begins with an evaluation of the reproducibility of the experimental method to establish reliability. Subsequently, the comparison between the calculated and experimentally determined delay zone lengths is presented, followed by the comparison of the experimentally determined melting lengths with the model-theoretically calculated melting lengths for the previously mentioned different values of a.

### 3.1. Reproducibility of the Results

**Figure 11:**
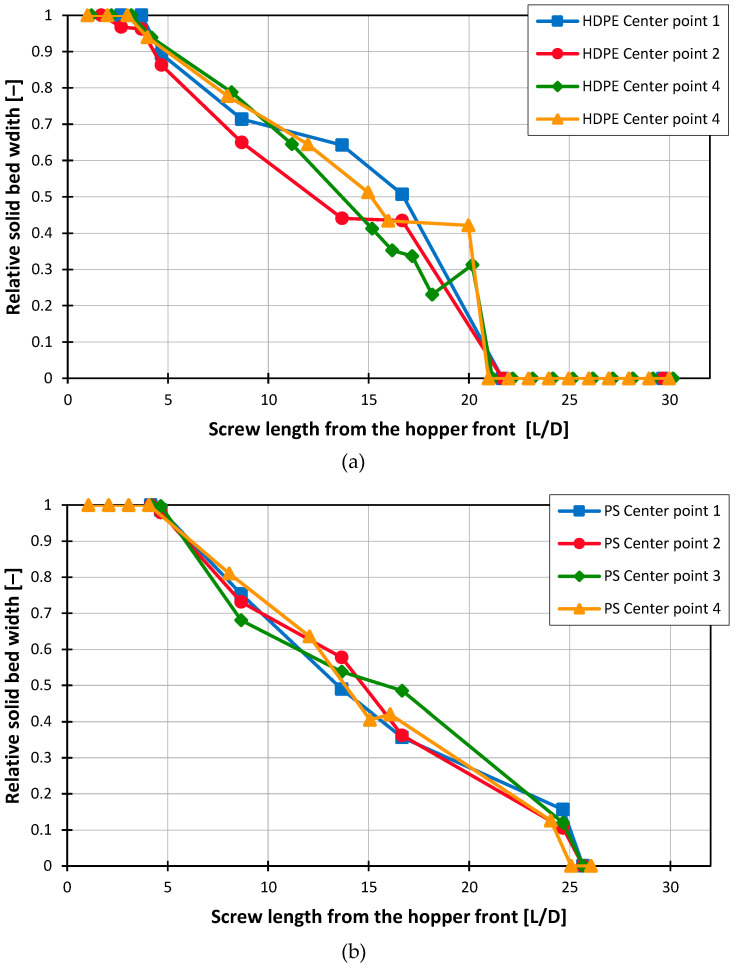
Melting profiles at 120 rpm, 2 mm pellet diameter, 200 °C barrel temperature using Screw 2 for (**a**) HDPE and (**b**) PS.

To assess the reproducibility of the results, the central point from the experimental design was repeated four times using Screw 2 for both materials. The results of the measured solid bed widths are shown in Figure 11. For illustration purposes, linear interpolation was applied between the individual measurement points in the diagram.

Since the model-based calculation excludes the region beneath the hopper—where full contact with the barrel has not yet been established—the positions for cross-sectional sample extraction were referenced from the hopper’s front edge. This approach ensures comparability between experimentally determined and calculated results. It was observed that, particularly for HDPE, variations in solid bed widths occurred along the screw, while both the starting point and the end of melting remained nearly identical across trials. For HDPE, the four measurements yielded an average standard deviation of 0.039 across all measurement points of the relative solid bed width, whereas PS demonstrated a slightly lower average standard deviation of 0.032.

Table 4 provides the average throughputs, delay zone lengths, and melting lengths from the investigations, along with the corresponding standard deviations, based on four repetitions of the central points for each material. The uncertainty in determining the delay zone length is ±0.5 L/D, while the uncertainty in determining the melting length is ±1 L/D.

### 3.2. Model Validation Regarding the Delay Zone Length

**Figure 12:**
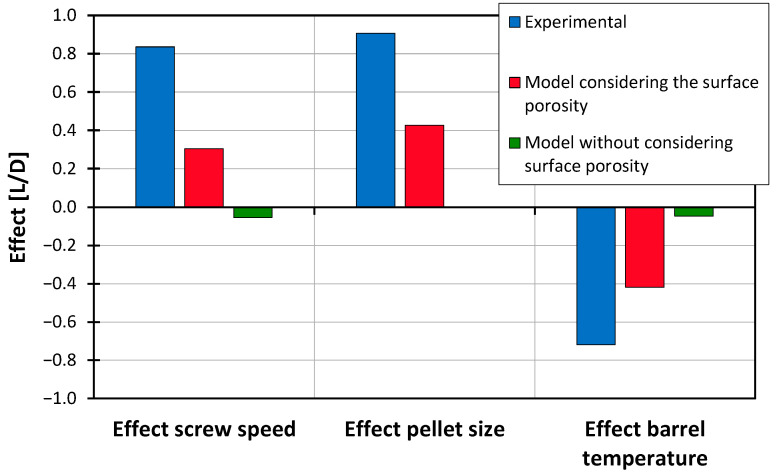
Comparison of the effects on the delay zone length between experiment and models.

This section presents the results from the comparison between the model-calculated delay zone lengths, based on the model described in Section 3.2, and the experimentally determined delay zone lengths. The comparison first examines the observed and calculated effects, with factor effects calculated according to [47]. The effects are determined by calculating the difference in mean values of the target variables at factor levels −1 and 1, based on the experimental design outlined in Table 2. In Figure 12, the effects of the investigated factors are shown. The data reveal that both screw speed and pellet size contribute to an increase in delay zone length, while higher barrel temperatures reduce it.

**Figure 13:**
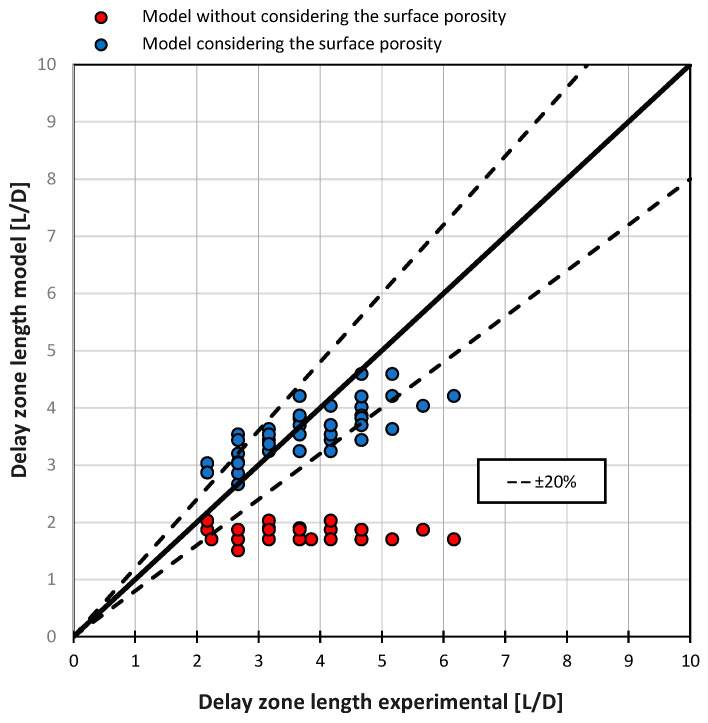
Comparison of experimentally determined and model-calculated delay zone lengths, both with and without consideration of surface porosity.

These effects are not accurately captured by models that exclude surface porosity. However, when surface porosity is incorporated, the model aligns more closely with the experimental observations, though the effects are somewhat more pronounced in the experiments. Figure 13 illustrates this comparison, presenting delay zone lengths as determined experimentally alongside model-calculated values, both with and without the inclusion of surface porosity.

The mean absolute error (MAE) for the model without accounting for surface porosity is 1.79 L/D, while incorporating surface porosity reduces the MAE to 0.54 L/D. This improvement demonstrates that the model’s accuracy significantly benefits from including surface porosity, bringing the calculated delay zone lengths into closer alignment with the experimental data.

### 3.3. Model Validation Regarding the Solid Bed Dynamics

To evaluate whether assuming specific solid bed acceleration or compression values improves model accuracy, experimentally determined melting profiles are compared with model-calculated melting profiles across different values of the parameter a. The following section illustrates the effect of varying a on calculated melting profiles, using representative examples.

**Figure 14:**
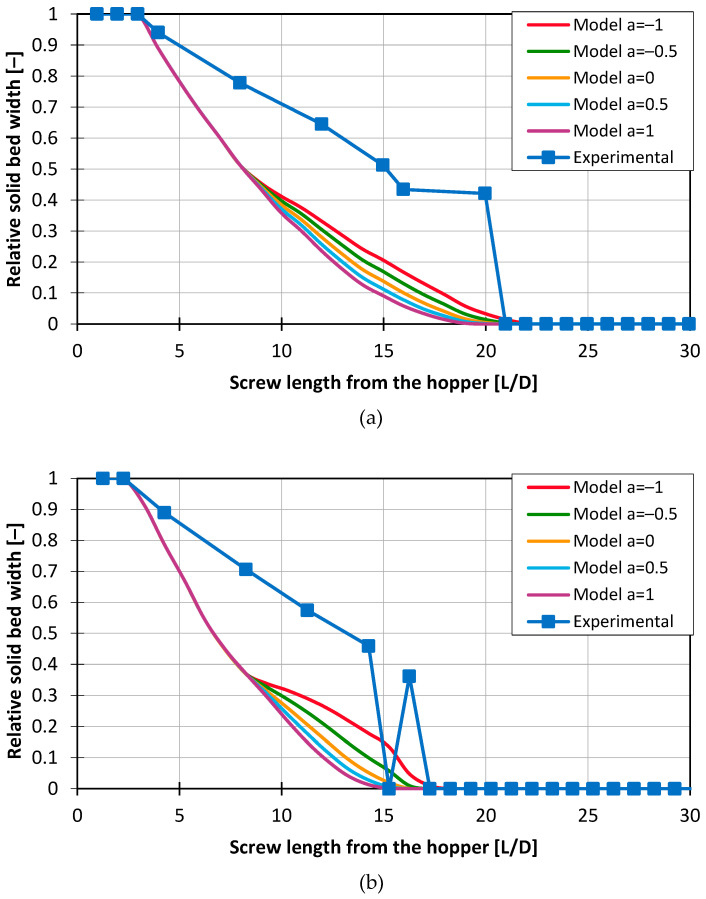
Melting profiles for HDPE at 120 rpm, 2 mm pellets, and medium back pressure for (**a**) Screw 2 and (**b**) Screw 3.

Figure 14 displays the melting profiles for the central experimental point using HDPE, comparing results for Screw 2, with a long compression zone, and Screw 3, with a short compression zone.

The results indicate that, for both processes, the model overestimates the melting rate at the beginning while significantly underestimating it toward the end. This behavior aligns with observations made by Tadmor in [15] for his analytical model. None of the variations in the parameter a can accurately represent this melting behavior. Additionally, in Figure 14b, a break-up of the solid bed at 15 L/D can be observed. This phenomenon will be discussed in more detail in the subsequent section.

**Figure 15:**
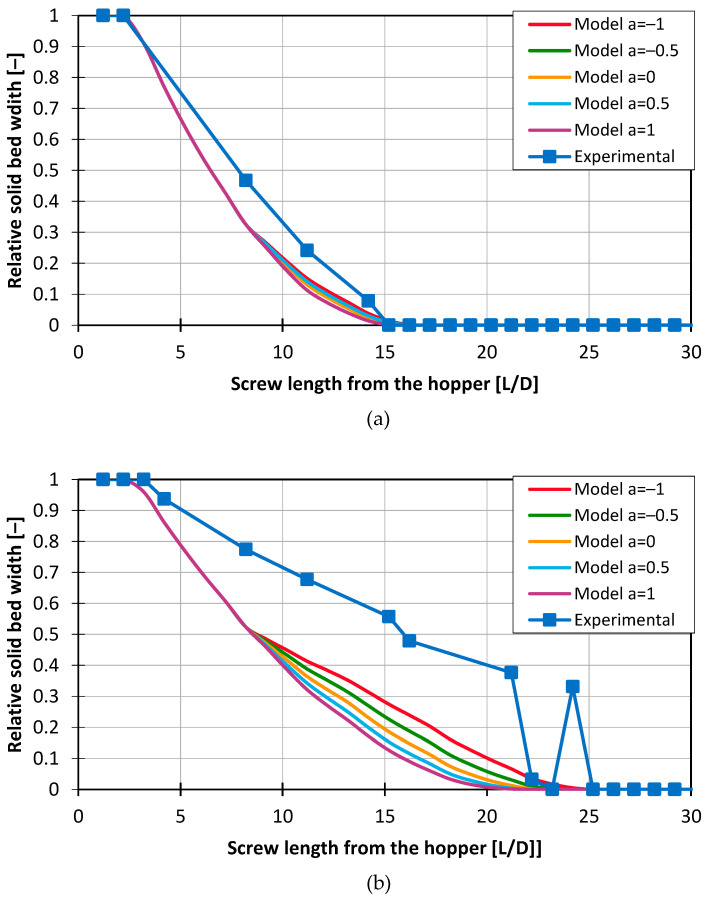
Melting profiles for HDPE with Screw 2, 2 mm pellets, and medium back pressure for (**a**) 60 rpm and (**b**) 180 rpm.

The discrepancy between the calculated and modeled melting profiles increases with rising screw speed, as shown in Figure 15. At a screw speed of 60 rpm, the model shows good agreement with the experimental data. However, at higher speeds, such as 180 rpm, the deviation becomes more pronounced.

This trend was consistently observed across all investigation points. Adjusting the model by varying the exponent a to account for changes in solid bed dynamics did not lead to any significant improvement in the model. However, the model reliably predicts the end of melting in most cases, regardless of the exponent a used.

Table 5 provides a summary of the mean absolute error (MAE), mean squared error (MSE), and maximum absolute error (MaxAE) across all investigation points, comparing the experimentally determined and model-calculated melting lengths under different assumptions for a.

The results indicate that no specific value of a provides a consistently clear advantage in minimizing model error. The lowest error metrics in this investigation were observed with a=0. However, the improvements are minor and fall within the measurement uncertainty of ±1 L/D for determining the end of melting. Thus, while a=0 yields slightly better results, the differences are not statistically significant and do not strongly support favoring one particular value of a over others.

#### Occurrence of Solid Bed Break-Ups

**Figure 16:**
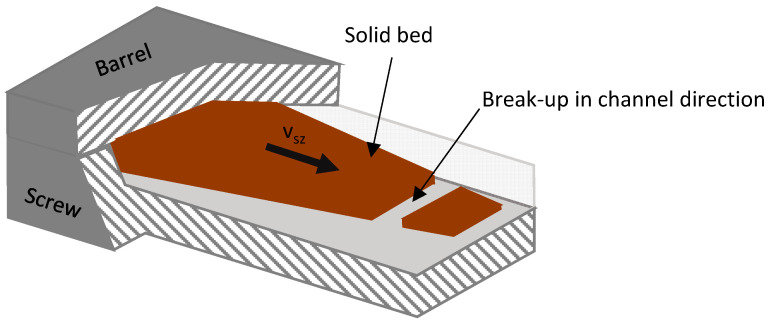
Schematic representation of a solid bed break-up [48].

In addition to the simplified model assumptions, another possible cause for the discrepancies between the experimental results and the model predictions is the solid bed break-up, as described by Tadmor in [15]. Solid bed break-up occurs along the channel direction, resulting in fully melt-filled sections within the melting zone. This phenomenon is schematically illustrated in Figure 16.

**Figure 17:**
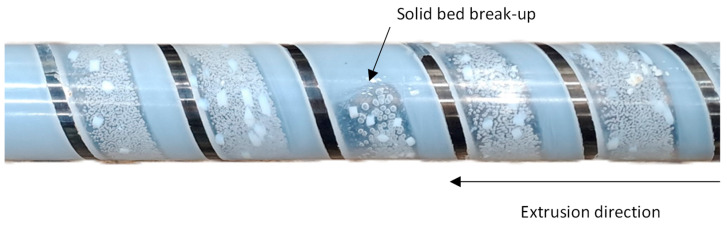
Solid bed break-up in PS observed after screw pull-out.

Solid bed break-ups were observed in these investigations with both materials and across all screws. For polystyrene, these break-ups were directly visible after the screw-pull experiment, as shown in Figure 17.

**Figure 18:**
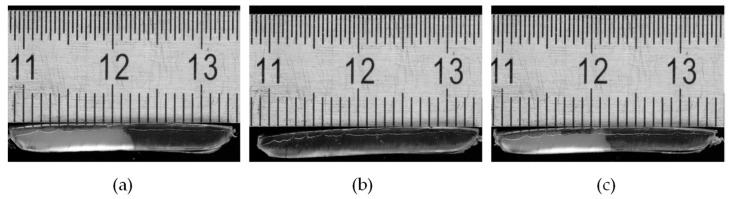
Cross-sectional samples at the position of a solid bed break-up: (**a**) before the break-up, (**b**) melt-filled channel, and (**c**) after the break-up.

Solid bed break-ups occurred exclusively in the compression and metering zones across all experiments and became more frequent with increasing screw speed. Regardless of the compression zone length, the break-ups usually occurred toward the end of the compression zone and the beginning of the metering zone. For Screw 3, with the shorter compression zone, the break-ups were frequently observed starting at a screw speed of 120 rpm. In contrast, with Screw 2 and its longer compression zone, break-ups were only detected at speeds of 180 rpm and above. In some cases, up to four solid bed break-ups were identified. Figure 18 shows cross-sectional samples taken at the position of a solid bed break-up.

It was observed that a melt film remains present immediately before the break-up, which contradicts Fukase’s “dam-up” phenomenon described in [30]. In that explanation, the disappearance of the melt film and subsequent direct contact between the solid bed and the barrel are suggested as potential causes of break-up, an assertion that could not be confirmed here. Additionally, unlike the findings in [48], no distinct melt film was detected at the screw root. This discrepancy suggests that the pressure gradient in the melt film beneath the solid bed, previously cited as a cause for the break-up, cannot be substantiated by the current results. Another potential factor could be Tadmor’s “surging” effect, which involves temporal fluctuations in temperature, pressure, and flow rate at the die, along with the entrapment of air bubbles in the melt stream [49]. An additional explanation for solid bed break-ups in the metering sections, also observed in this study, is provided by Chung. The melt pool flowing directly adjacent to the solid bed in these zones could exert stress on the decelerating solid bed, potentially leading to break-up [1].

However, based on the data generated in this study, none of the existing explanations in the literature for the occurrence of solid bed break-ups can be clearly confirmed or refuted, highlighting the need for further investigation of this phenomenon in future studies.

## 4. Discussion

This paper introduced a range of theoretical models for describing the melting process, including analytically solvable models with simplifying assumptions and others that require numerical solutions. Analytical models offer advantages in ease of use and practical applicability for engineers. Consequently, this study revisited and validated the Tadmor model modified by Potente, which enables a comprehensive description of the melting process. Specific model parameters were redefined based on the experimentally determined data.

In particular, the correct determination of the flow temperature at the phase boundary between the solid bed and the melt proved to be a decisive factor in improving the model’s accuracy. Since the flow temperature for amorphous thermoplastics cannot be precisely determined, it was assumed to be 50 °C above the melting temperature in this study. For semi-crystalline thermoplastics, the flow temperature was chosen to be at the end of the crystalline melting range rather than at the DSC peak temperature.

To better predict the delay zone length as described by Tadmor, surface porosity was incorporated into the model. Given the challenges of analytically describing surface porosity due to multiple influencing factors, a simplified approach was employed, calculating surface porosity based on the more accessible bulk porosity. This modification significantly enhanced the model’s prediction accuracy for delay zone length.

Additionally, the parameter a, which describes the solid bed dynamics in zones with varying channel depth, was analyzed by comparing experimentally determined melting profiles and model-calculated melting profiles across different values of a. No significant improvement in the model was found by accounting for specific solid bed acceleration or compression. However, this does not imply that no variable solid bed dynamics occur in the real process. It was shown that the assumption of a constant temperature profile in the solid bed does not allow for an accurate description of the melting rate along the screw channel. The effect of variable solid bed dynamics on the model is too small to result in significant improvement. Since the best agreement between the model and the experimental investigations was found for the assumption a=0, this value will be retained for the model.

**Figure 19:**
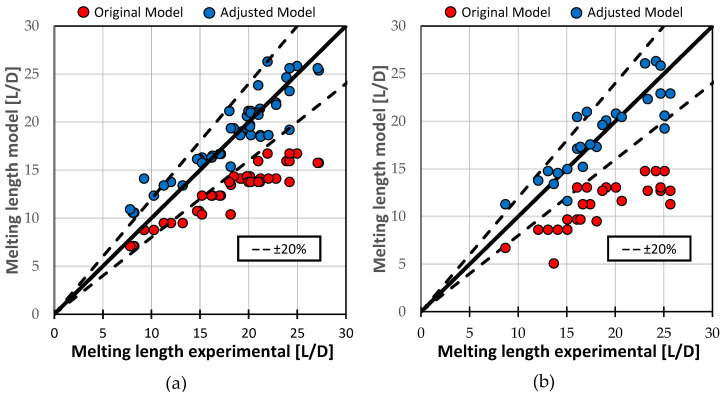
Comparison of experimentally determined and model-theoretically calculated melting lengths for (**a**) HDPE and (**b**) PS.

Nevertheless, the adjusted analytical model provided good predictions of the melting endpoint across all processes. Figure 19 compares the original model and the adjusted model against the experimental results for melting length. For HDPE, the mean absolute error decreased from 5.36 L/D with the original model to 1.52 L/D with the adjusted model. For PS, the improvement was even more pronounced, with the mean absolute error reducing from 8.04 L/D in the original model to 1.44 L/D in the adjusted model.

One phenomenon that complicates the prediction of melting profiles is solid bed break-up along the channel direction, which was frequently observed in this study. Existing explanations from the literature could not be clearly confirmed, making this phenomenon a subject for further investigation in future studies.

## Figures and Tables

**Table 1:** Geometry of the utilized screws.

Geometric Aspect	Screw 1	Screw 2	Screw 3
Diameter [mm]	45	30	30
Length [L/D]	32.9	35.4	35.4
Pitch [L/D]	1	1	1
Flight width [mm]	5	3	3
*Feed Section*			
Length [L/D]	13.9	12.7	12.7
Channel depth [mm]	9	5.4	5.4
*Compression section*			
Length [L/D]	11	16	7
*Metering section*			
Length [L/D]	8	6.7	15.7
Channel depth [mm]	3.5	2.1	2.1

**Table 2:** Experimental plan for the investigation of delay zone length.

Factor	Unit	−α	−1	0	+1	+α
Barrel temperature	°C	170	180	200	220	230
Screw speed	rpm	30	60	120	180	210
Pellet diameter	mm	-	1	2	3	-

**Table 3:** Experimental plan for the investigation of solid bed dynamics.

Factor	Unit	−α	−1	0	+1	+α
Rotational Speed	rpm	30	60	120	180	210
Pellet diameter	mm	-	1	2	3	-
Back pressure	-		Low	Medium	High	

**Table 4:** Comparison of throughput, delay zone length, and melting length.

Experimental Point	Throughput [kg/h]	Delay Zone Length [L/D]	Melting Length [L/D]
*HDPE*			
Average	15.54	3.88	21.5
Standard deviation	0.12	0.25	0.58
*PS*			
Average	20.16	4.38	25.75
Standard deviation	0.17	0.25	0.5

**Table 5:** Comparison of MAE, MSE, and MaxAE for different values of a in the prediction of melting length for HDPE and PS.

Variations	MAE [L/D]	MSE [L²/D²]	MaxAE [L/D]
*HDPE*			
a = −1	2.40	7.80	7.56
a = −0.5	1.71	4.16	4.90
a = 0	1.52	3.85	5.00
a = 0.5	1.61	4.36	6.08
a = 1	1.94	5.82	6.80
*PS*			
a = −1	1.78	8.35	6.61
a = −0.5	1.62	5.97	6.01
a = 0	1.44	4.98	5.83
a = 0.5	1.37	5.16	6.79
a = 1	1.66	7.54	7.17

## Data Availability

Data are contained within the article.

## Nomenclature

**Abbreviations**	
**Character**	**Meaning**
CFD	Computational Fluid Dynamics
DEM	Discrete Element Method
DSC	Differential Scanning Calorimetry
FEM	Finite Element Method
FVM	Finite Volume Method
HDPE	High Density Polyethylen
KTP	Kunststofftechnik Paderborn
MAE	Mean Absolute Error
MaxAE	Maximum Absolute Error
MSE	Mean Squared Error
PS	Polystyrene
**Roman characters**	
**Character**	**Meaning**
a	Exponent for consideration of the solid bed dynamics
a_S_	Thermal diffusivity of the solid
c	Contour exponent of the melt film
c′	Contour exponent of the melt film taking into account the leakage flow
D	Barrel diameter
d_p_	Pellet diameter
E	Empirically determined exponent
E_r_	Relaxation modulus
h	Screw channel height
h_sb_	Solid bed height
k1	Parameter to take into account the temperature dependence of viscosity
L	Length
${\dot{q}}_{sb}$	Heat flux into the solid bed
T_fl_	Flow temperature
T_g_	Glass transition temperature
T_m_	Crystallite melting temperature
T_s_	Temperature of the solid
u	Normalized height of the solid bed
v_0_	Cylinder circumferential speed
v_0x_	Cylinder circumferential speed in x-direction
v_0z_	Cylinder circumferential speed in z-direction
v_sz_	Velocity of the solid bed in z-direction
w	Channel width
Y	Normalized solid bed width
Z	Length of the channel interval
**Greek characters**	
**Character**	**Meaning**
α	Axial point factor
β	Slope parameter
δ	Melt film thickness
δ_0_	Melt film thickness at the active flight
${\bar{\delta }}_{AS}$	Average melt film thickness of the melting zone
Δh_m_	Melting enthalpy
Δh_s_	Solid enthalpy
ε	Surface porosity
ε_s_	Proportion of contact surface between granulate and cylinder
η	Shear viscosity
λ_s_	Thermal conductivity of the melt
π	Normalized melting rate
ρ_b,0_	Density of the bulk material
ρ_b,channel_	Density of the bulk material in the screw channel
ρ_m_	Density of the melt
ρ_s_	Density of the solid
φ	Helix angle
Ψ	Normalized melt film thickness
Ψ_S_	Normalized melt film thickness over the flights
